# Frequency and genetic spectrum of maturity-onset diabetes of the young (MODY) in southern New Zealand

**DOI:** 10.1186/2251-6581-12-46

**Published:** 2013-12-19

**Authors:** Benjamin J Wheeler, Nicola Patterson, Donald R Love, Debbie Prosser, Paul Tomlinson, Barry J Taylor, Patrick Manning

**Affiliations:** 1 Department of Women’s and Children’s Health, University of Otago, Dunedin, New Zealand; 2 Edgar National Centre for Diabetes and Obesity Research, University of Otago, Dunedin, New Zealand; 3 Diagnostic Genetics, LabPlus, Auckland, New Zealand; 4 School of Biological Sciences, University of Auckland, Auckland, New Zealand; 5 Department of Endocrinology, Dunedin Public Hospital, Dunedin, New Zealand

**Keywords:** MODY, Diabetes, Paediatrics, Monogenic Diabetes, Genetics

## Abstract

Maturity-Onset Diabetes of the Young (MODY) is a monogenic form of diabetes, consisting of a heterogeneous group of autosomal dominant inherited disorders. Typical onset is in individuals prior to twenty five years, and presentation can mimic type 1 or 2 diabetes. Molecular genetic testing can allow precise identification of the different MODY sub-types. Making a specific diagnosis of MODY can have important implications for the guidance of appropriate treatment, prognosis and genetic counselling.

We present the cases of three children and their families diagnosed with MODY over the past two years. These families highlight the features of three of the more common MODY subtypes, including two with novel mutations, one of which segregates in a kindred that is strongly affected by both MODY and classic autoimmune mediated diabetes. To date, we have identified a prevalence of MODY in the paediatric diabetes population of the lower South Island, New Zealand, of approximately 2.5%. This prevalence, along with increasing access to molecular genetic testing, highlights the importance of consideration of MODY in atypical diabetes presentations in the paediatric/adolescent population.

**Electronic supplementary material:**

The online version of this article (doi:10.1186/2251-6581-12-46) contains supplementary material, which is available to authorized users.

## Background

Maturity Onset Diabetes of the Young (MODY) is a monogenic form of diabetes. It consists of a heterogeneous group of autosomal dominant inherited disorders, with typical onset in individuals aged less than twenty five years [1, 2]. There are several sub-types of MODY which can be precisely identified using molecular genetic testing, the four most common of which [3] are outlined in Table 1 (in descending order of frequency). Modes of presentation vary and can mimic either type 1 or 2 diabetes. Making a specific diagnosis of MODY can have important implications for the guidance of appropriate treatment, prognosis and genetic counselling.

**Table 1:** **MODY subtypes (4 most common in descending order of frequency)**

Gene and MODY subtype	***Gene function***+ phenotype	Prognosis
HNF1-alpha gene	*Regulates insulin gene transcription*	Progressive
(MODY 3)	Reduced insulin secretion/diabetes and marked sensitivity to sulfonylurea	May require insulin
		May develop secondary complications
Glucokinase (GCK) gene	*Catalyses conversion of glucose to glucose-6-phosphate*	Generally non or slowly progressive
(MODY 2)	Reduced glucose sensing by beta cells – Mild diabetes	Complications rare
HNF4-alpha gene	*Nuclear transcription factor that regulates hepatic and pancreatic beta cell gene expression*	Progressive
(MODY 1)	Reduced insulin secretion/diabetes and marked sensitivity to sulfonylurea	May require insulin
		May develop secondary complications
HNF1-beta gene	*Regulates HNF4á gene transcription*	Progressive beta-cell failure with diabetes onset around puberty
(MODY 5)	Insulin resistance + wide clinical spectrum	Insulin resistance without obesity
	+/- Urogenital/pancreatic anomalies	Insulin dependence
	+/- Pancreatic exocrine failure	
	+/- Developmental delay/Learning difficulties	

The Southern District Health Board paediatric diabetes team provides sole care for approximately 160 children and adolescents spread over the largest geographical region in New Zealand. In this report we present three children and their families who have been diagnosed with MODY over the past two years. We identified two novel mutations and a kindred strongly affected by both MODY and classic autoimmune-mediated diabetes.

## Case presentation

### Case 1

**Figure 1:**
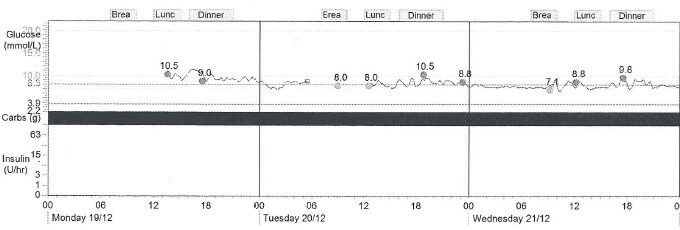
**Typical BGLs on continuous glucose monitoring - case 1.**

**Figure 2:**
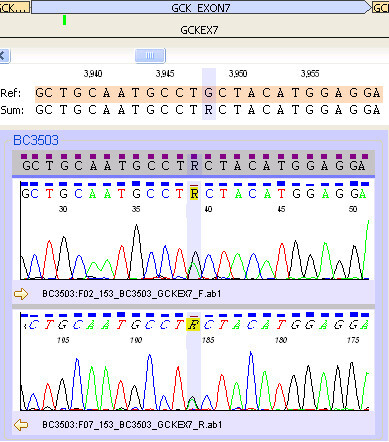
**One copy of the variant c.698G>A (p.Cys233Tyr) in exon 7 of the** ***GCK*** **gene (Refseq accession number NM_000162).**

**Figure 3:**
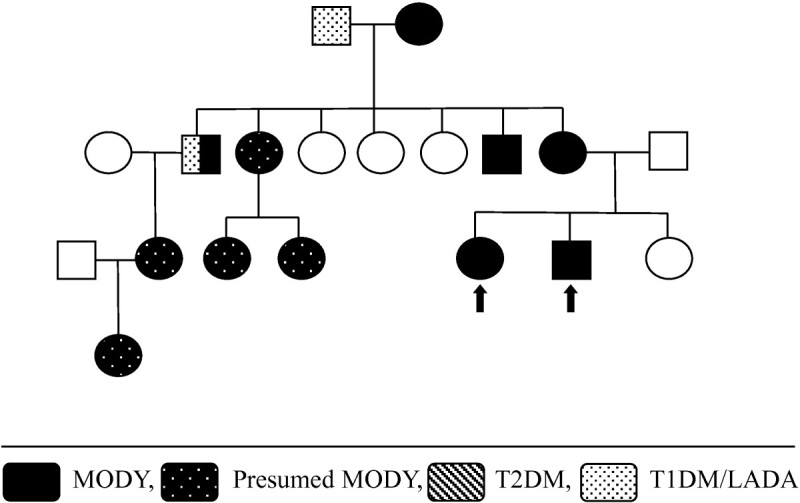
**Family tree - case 1.**

A 9 year old Caucasian boy with a BMI of 19 kg/m^2^ (z-score 1.5) presented in 2006 with mild diabetes (two independent fasting blood glucose levels (BGL) >7.0 mmol/L), and was both autoantibody-negative and non-insulin resistant, requiring no immediate treatment. He was initially lost to follow up, but represented in 2010 aged 13 years, along with his sister aged 11 years. Both were asymptomatic but had on-going mild hyperglycaemia (Figure 1). The persistent mild hyperglycaemia, combined with a very significant family history of early onset type two diabetes (T2DM), led to gene screening for a glucokinase (*GCK*) gene abnormality (MODY 2 subtype). Complicating matters were family members with proven antibody positive (glutamate decarboxylase (GAD) positive) type one diabetes (T1DM), with associated complications. A novel mutation was subsequently identified (Figure 2) in exon 7 of the glucokinase (*GCK*) gene, in multiple individuals from this kindred (Figure 3). A maternal uncle with latent autoimmune diabetes (LADA) (GAD positive) was identified with this mutation. These children remain well without treatment. Regarding case one, his HbA1c is currently 42 mmol/mol (6%).

### Case 2

**Figure 4:**
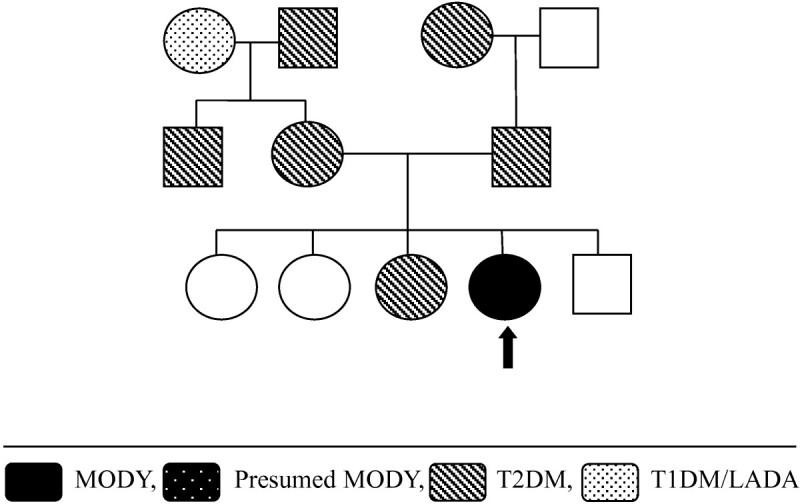
**Case 2 - Family tree.**

A 14 yr old Cook Island Maori girl with a BMI of 23 kg/m^2^ (z-score 1.1) presented with severe non-ketotic hyperglycaemia (BGL 71.7 mmol/L (NR 4–6)) and hypernatraemic dehydration (corrected Na 150 mmol/L (135–145), serum osmolality 364 mosm/kg (275–295)), pH 7.39, and lactate of 5.3 mmol/L (NR 0.5-2). There was evidence of insulin resistance with fasting insulin 336 pmol/L (10–80), C-peptide 1180 pmol/L (350–750), and clinical acanthosis nigricans. Insulin autoantibodies were negative. She had moderate, unexplained intellectual disability with some subtle dysmorphic facial features. There was an extensive family history of T2DM (Figure 4). Initial treatment consisted of insulin up to 3 units/kg/day. A microarray study (Agilent ISCA (v2) 60 K whole genome array) demonstrated a novel 1.3 Mb deletion at chromosome 17q12, this segment includes the *HNF1β* and multiple other genes. Thus, there is a haplo-insufficiency of *HNF1β*. Parental studies were normal, showing this to be a de novo deletion. Mutation within the *HNF1β* gene may cause urogenital abnormalities as well as MODY, but renal and pelvic ultrasonography were normal. The intellectual disability and subtle facial dysmorphism may also be due to the loss of other genes within this deleted segment. Currently her HbA1c is 48 nmmol/mol (6.5%) on insulin 1.25 units/kg/day and 500 mg TDS Metformin.

### Case 3

**Figure 5:**
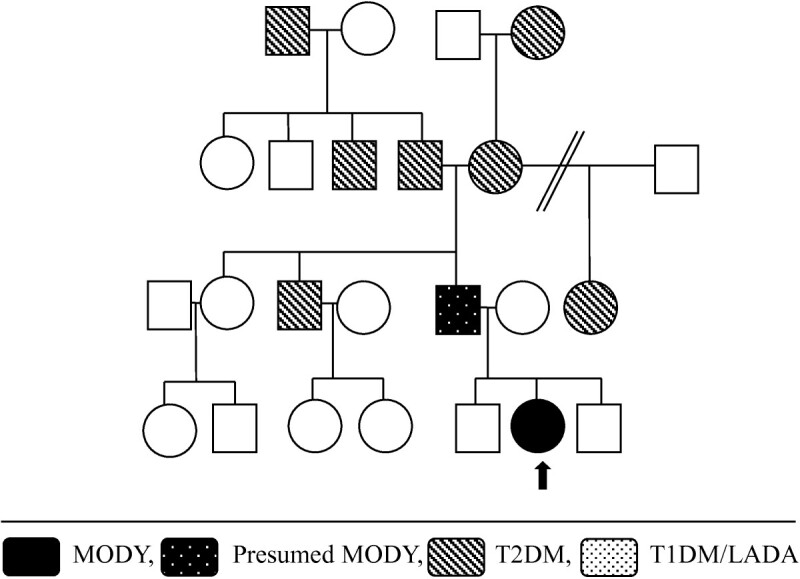
**Case 3 – family tree.**

**Figure 6:**
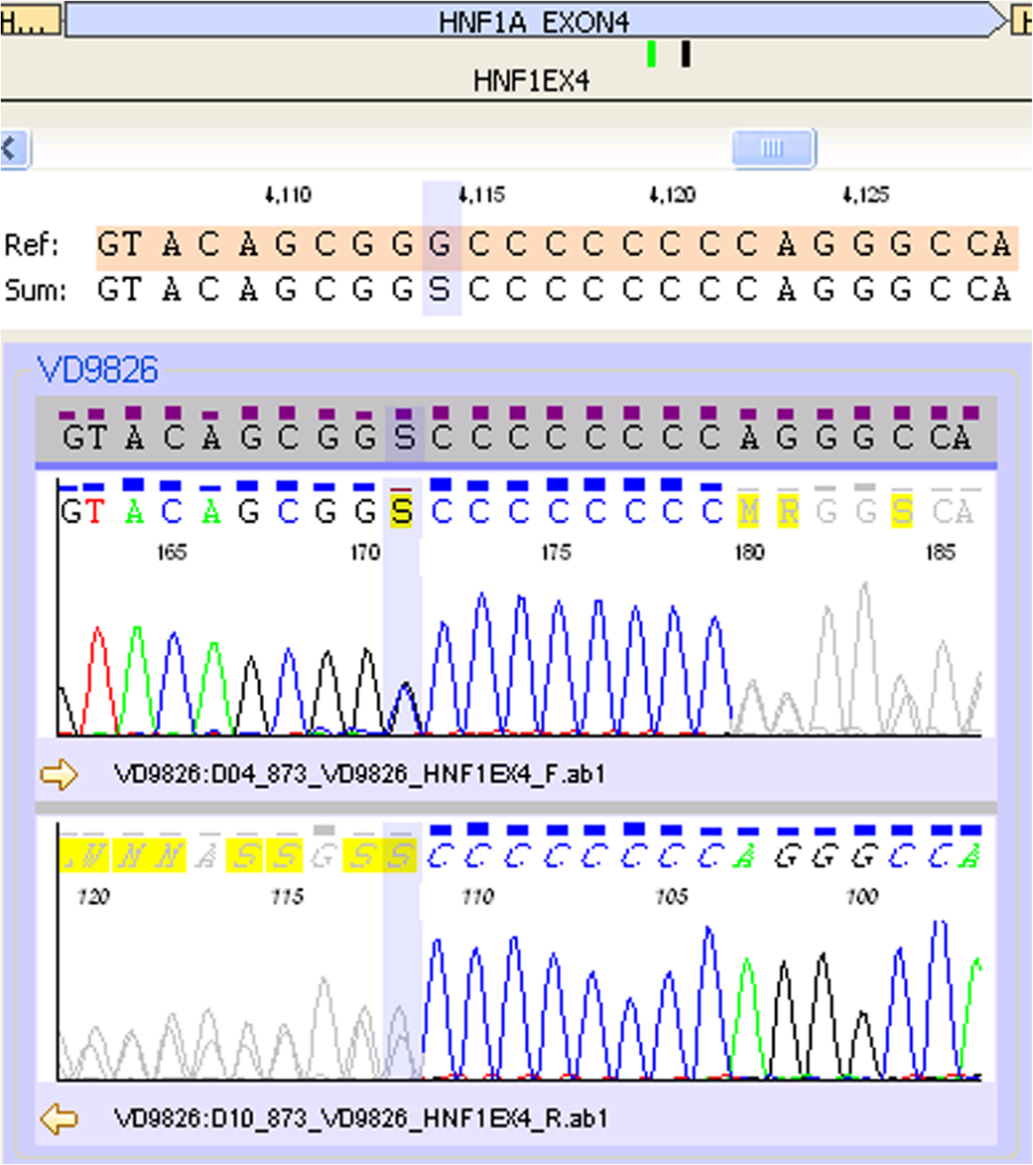
**Frameshift mutation c.864delGinsCC, or c.864G>C and c.872dupC, (p.Gly292ArgfsX25) in exon 4 of the** ***HNF1α*** **gene (Refseq accession number NM_000545).**

An 11 yr old Caucasian girl with a BMI of 16 kg/m^2^ (z-score -1.2) presented with a 4 month history of recurrent mucosal candidiasis and mild postprandial hyperglycaemia. Diabetes was confirmed with two random BGLs >11.1 mmol/l. She was well on presentation with a blood pH 7.33, and negative for ketones. Diabetes autoantibody screening was negative, as were clinical and biochemical signs of insulin resistance. Insulin was commenced but her requirement was low at 3 units of isophane daily with aspart as needed with meals (<2 units/day). A significant family history was uncovered (Figure 5) and an *HNF1α* (MODY 3) gene mutation was suspected and subsequently confirmed on molecular genetic testing (Figure 6). Currently, she is well controlled on Gliclazide 80 mg *mane* with an HbA1c of 51 mmol/mol (6.8%).

## Discussion

In the past two years we have identified four children and their families with MODY, covering three subtypes. In each of these cases the diagnosis of MODY has been confirmed by molecular genetic testing. We have identified two novel gene mutations, one in the *GCK* gene (MODY 2), and one in the *HNF1β* gene (MODY 5). Clinically, there is diverse range of presentation for MODY subtypes, with our cases highlighting some of this phenotypic variation.

Making a diagnosis of MODY has important implications for treatment, prognosis, and genetic counselling. Glucokinase gene mutations (MODY 2) often require no treatment [1, 2]. In case one, making this diagnosis has led to: reassurance; reduced monitoring; re-diagnosis of many family members; and subsequent acceptance of no treatment for most. *HNF1α* (MODY 3) and *HNF4α* (MODY 1) gene mutations usually have a marked sensitivity to oral sulfonylureas [1, 2, 4]. For case three this allowed for cessation of insulin therapy. Unfortunately, as in the father of this patient, an insulin requirement often develops as the disease progresses [5]. In case two, diagnosing an *HNF1β* (MODY 5) gene deletion led to an explanation of a previously unexplained phenotype, as well as guidance for screening for associated abnormalities.

Case one also highlights the rare occurrence of a kindred that is strongly affected by both MODY and classic autoimmune mediated diabetes. One member of this family now has diabetes consistent with LADA (GAD +ve) combined with the novel *GCK* gene mutation. This is extremely rare, occurring in <1% of MODY cases [6].

To date (based on these four cases) the Otago/Southland regions in the south of New Zealand have a paediatric MODY prevalence of 2.5% in children with diabetes. While this is based on very small numbers, there is no other data currently published for New Zealand or Australia on MODY prevalence. While similar to the 2.4% paediatric rate reported by Galler et al. [7] for Saxony (Germany), this appears high compared to some worldwide clinic reports [8–10]. Given current estimates of prevalence in adult diabetes of 2-5% [1], it is likely that many MODY cases remain undiagnosed in both paediatric and adult clinics [3, 11]. Vigilance should remain particularly for those with two or more atypical clinical features, such as: no features of insulin resistance; negative β cell autoimmunity; non-ketotic presentations; a strong family history of diabetes (any type); no or unusually low insulin requirement; onset prior to 25 years of age; and unusual or atypical associated phenotypes [1, 2, 4].

## Conclusions

In conclusion, our local prevalence, along with increasing access to molecular genetic testing, highlights the importance of considering MODY in atypical diabetes presentations in the paediatric and adolescent diabetes populations.

## Consent

Written informed consent was obtained from the patients and the patient’s legal guardians, for publication of this case report and accompanying images. A copy of the written consent is available for review by the Editor-in-Chief of this journal.

## Authors’ original submitted files for images

Below are the links to the authors’ original submitted files for images. Authors’ original file for figure 1 Authors’ original file for figure 2 Authors’ original file for figure 3 Authors’ original file for figure 4 Authors’ original file for figure 5 Authors’ original file for figure 6

## Abbreviations

MODY: Maturity onset diabetes in young
BMI: Body mass index
T2DM: Type 2 Diabetes Mellitus
GCK: Glucokinase
HNF1α: hepatocyte nuclear factor 1-alpha gene
GAD: Glutamate decarboxylase
T1DM: Type 1 Diabetes Mellitus
LADA: Latent autoimmune diabetes of adults
CGMS: Continuous glucose monitoring system
HNF4α: Hepatocyte nuclear factor 4-alpha gene
NR: Normal range
TDS: Three times daily
HNF1β: Hepatocyte nuclear factor 1-beta gene.
