# Supplementary figures and images for: Frequency and genetic spectrum of maturity-onset diabetes of the young (MODY) in southern New Zealand
Source: J Diabetes Metab Disord. 2013 Dec 19;12:46. doi: 10.1186/2251-6581-12-46 (PMC7963407; doi:10.1186/2251-6581-12-46)

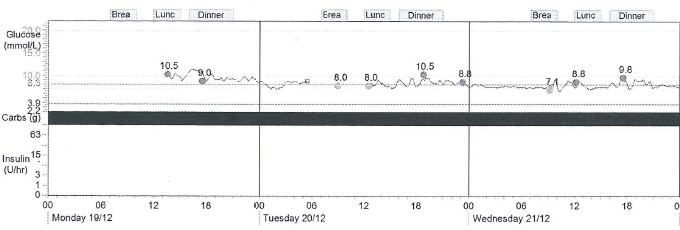

Supplement: Supplementary file 1 — Authors’ original file for figure 1 [file 40200_2013_178_MOESM1_ESM.png]

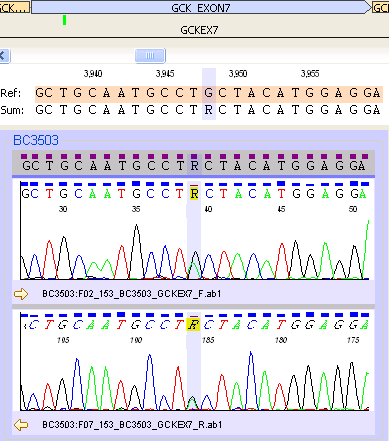

Supplement: Supplementary file 2 — Authors’ original file for figure 2 [file 40200_2013_178_MOESM2_ESM.png]

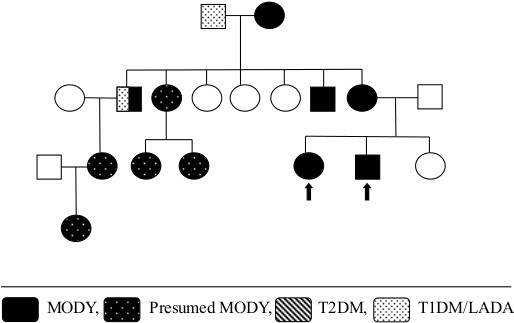

Supplement: Supplementary file 3 — Authors’ original file for figure 3 [file 40200_2013_178_MOESM3_ESM.jpeg]

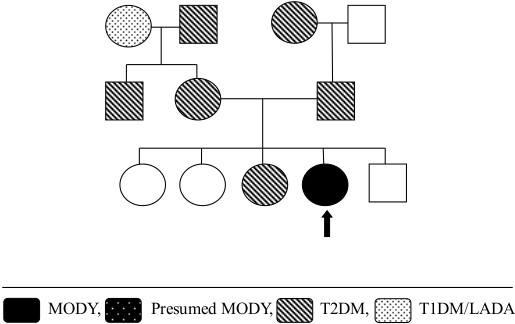

Supplement: Supplementary file 4 — Authors’ original file for figure 4 [file 40200_2013_178_MOESM4_ESM.jpeg]

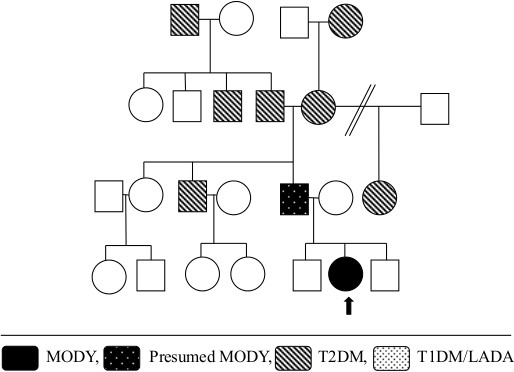

Supplement: Supplementary file 5 — Authors’ original file for figure 5 [file 40200_2013_178_MOESM5_ESM.jpeg]

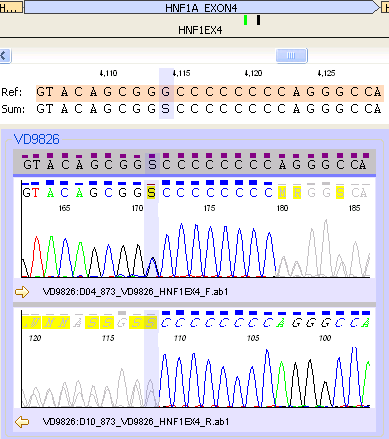

Supplement: Supplementary file 6 — Authors’ original file for figure 6 [file 40200_2013_178_MOESM6_ESM.png]
